# Epigenetic Biomarkers for the Diagnosis and Treatment of Liver Disease

**DOI:** 10.3390/cancers13061265

**Published:** 2021-03-12

**Authors:** María Arechederra, Miriam Recalde, María Gárate-Rascón, Maite G. Fernández-Barrena, Matías A. Ávila, Carmen Berasain

**Affiliations:** 1 Program of Hepatology, Center for Applied Medical Research (CIMA), University of Navarra, 31008 Pamplona, Spain; macalderon@unav.es (M.A.); mrecaldedom@alumni.unav.es (M.R.); mgarate.3@alumni.unav.es (M.G.-R.); magarfer@unav.es (M.G.F.-B.); 2 IdiSNA, Navarra Institute for Health Research, 31008 Pamplona, Spain; 3 National Institute for the Study of Liver and Gastrointestinal Diseases (CIBERehd, Carlos III Health Institute), 28029 Madrid, Spain

**Keywords:** precision medicine, DNA methylation, epidrugs, liquid biopsy, hepatocellular carcinoma, liver fibrosis

## Abstract

**Simple Summary:**

Cellular identity and function depends on the correct and specific expression of genetic information. Different epigenetic mechanisms including DNA methylation, modifications of histones, and expression of non-coding RNAs control chromatin structure and DNA accessibility to the transcriptional machinery. Alterations of these mechanisms are associated with the development of multiple diseases. The incidence of chronic liver diseases, including hepatocellular carcinoma (HCC), is increasing worldwide. However, the management of those patients is poor due to the late diagnosis of the disease and the absence of effective therapies. Here, we review the epigenetic alterations described in the process of hepatocarcinogenesis, and we discuss the applicability of epigenetic markers as therapeutic targets and liquid biopsy biomarkers for diagnosis.

**Abstract:**

Research in the last decades has demonstrated the relevance of epigenetics in controlling gene expression to maintain cell homeostasis, and the important role played by epigenome alterations in disease development. Moreover, the reversibility of epigenetic marks can be harnessed as a therapeutic strategy, and epigenetic marks can be used as diagnosis biomarkers. Epigenetic alterations in DNA methylation, histone post-translational modifications (PTMs), and non-coding RNA (ncRNA) expression have been associated with the process of hepatocarcinogenesis. Here, we summarize epigenetic alterations involved in the pathogenesis of chronic liver disease (CLD), particularly focusing on DNA methylation. We also discuss their utility as epigenetic biomarkers in liquid biopsy for the diagnosis and prognosis of hepatocellular carcinoma (HCC). Finally, we discuss the potential of epigenetic therapeutic strategies for HCC treatment.

## 1. Introduction

Although all the cells in a multicellular organism share a common genome, they display a specific phenotype. This heritable but dynamic cell identity depends on chromatin states established by epigenetic mechanisms [1]. Cell type–specific epigenomic landscapes or epigenomes are responsive to genetic, environmental, and metabolic cues and are associated with specific active and repressive chromatin regions that dictate DNA accessibility to the transcriptional machinery controlling gene expression and cellular states [2]. Epigenetic regulation of chromatin includes DNA methylation, nucleosome histone variants, post-translational histone modifications, and non-coding RNAs (ncRNAs) [1]. This epigenetic information is maintained through the coordinated activity of a plethora of epigenetic factors that modulate nucleosome structure and positioning (remodelers), establish epigenetic marks in DNA and histones (writers), recognize and interpret the marks (readers), and remove these marks (erasers) [3,4,5,6].

The epigenetic marks include the methylation of cytosine bases within CpG dinucleotides (5mC) in the DNA by DNA methyltransferases (DNMTs). The 5mC mark in CpG-rich promoter regions is associated with gene repression, and it is acquired in cancer cells to inhibit the expression of tumor suppressor genes [7]. On the contrary, the 5mC mark within gene bodies is linked to gene activation [8] and oncogene induction in tumors [9]. The presence of 5-hydroxymethylcytosine (5hmC) in DNA has been known since 1972 [10]. Oxidation of 5mC to 5hmC by the dioxygenase ten-eleven-translocation (TET) family of enzymes is now considered a stable epigenetic mark with regulatory functions in the genome that is significantly reduced in cancer [11,12,13,14].

DNA is wrapped around eight core histones (two copies of H2A, H2B, H3, and H4) forming the nucleosome, the basic unit of the chromatin. Histone-modifying enzymes add or remove covalent post-translational modifications (PTMs) including methylation, acetylation, phosphorylation, SUMOylation, ubiquitination, or ADP-ribosylation to the long *N*-terminal tail of histones, affecting chromatin packaging. For instance, histone acetylation as a result of positive lysine charge neutralization facilitates the opening of the chromatin. However, the effect of histone methylation depends on the targeted residues so that methylated H3K4/36/79 are considered active marks and methylated H3K9/27 and H4K20 repressive marks [15,16]. In general, chromatin conformation is changed through the specific binding of protein complexes to these PTMs, allowing the activation or repression of gene expression [17]. The epigenetic regulation of transcription also involves the activity of ncRNAs including microRNAs (miRNAs), PIWI-interacting RNAs (piRNAs), short-interfering RNAs (siRNAs), enhancer RNAs, and long non-coding RNAs (lncRNAs) acting through chromatin and post-transcriptional regulation (miRNAs) [1]. 

Chromatin stability is required to maintain cellular homeostasis; therefore, alternative chromatin states and epigenetic reprogramming are associated with a wide range of common diseases [1,2,18]. As mentioned above, the establishment of the corresponding epigenetic modifications of DNA and chromatin depends on the correct expression and function of a plethora of epigenetic factors, but their activity also depends on the availability of multiple metabolites involved in the different enzymatic reactions [19]. Consequently, metabolic alterations can induce global perturbations of the epigenome as well as alterations of specific loci [1,20]. Moreover, mutations affecting metabolic genes and the genes encoding remodelers, writers, readers, and erasers will contribute to the rewiring of the epigenomic landscape [1,20,21]. Reciprocally, mutation rates can be affected by DNA methylation [22] and nucleosome positioning [23]. Altogether, accumulating data demonstrate that cellular health requires an equilibrium between environmental, genetic, and epigenetic factors. Moreover, as epigenetic changes are reversible, reverting epigenetic and transcriptional reprogramming represents a therapeutic opportunity. 

Here, we will review recent discoveries regarding the epigenetic mechanisms involved in the pathogenesis of chronic liver disease (CLD) and the utility of epigenetic biomarkers in liquid biopsy for the diagnosis and prognosis of hepatocellular carcinoma (HCC). In both cases we will focus more specifically on DNA methylation. Finally, we will review the potential of epigenetic therapeutic strategies for HCC.

## 2. Epigenetic Reprogramming in Liver Disease: Changes in Epigenetic Marks and Modifiers

The burden of CLDs represents a major public health problem worldwide [24,25]. Etiological factors are well known and include hepatitis B virus (HBV), hepatitis C virus (HCV), long-lasting alcohol abuse, and obesity, insulin resistance, or diabetes leading to non-alcoholic fatty liver disease (NAFLD) [26,27]. The prognosis and management of a patient with CLD largely depends on the stage at which the disease is diagnosed, i.e., simple steatosis, hepatitis, fibrosis, cirrhosis, and cancer, and the progression through these stages is highly variable among individuals. Importantly, early stages of the disease could be reversible; however, therapeutic options are limited for cirrhotic patients, in which most cases of HCC occur [28]. The prognosis of HCC patients remains poor, and their survival largely depends on early diagnosis. However, almost half of HCC patients are diagnosed at an advanced stage [27]. In this scenario it is urgent to identify biomarkers of CLD progression as well as effective therapeutic targets.

In this sense, great efforts have been made to better understand the molecular and cellular mechanisms implicated in the progression of CLD. Over the last decade, next-generation sequencing technologies have been used to identify the most frequent mutations, DNA copy variations, and changes in gene expression that contribute to hepatocarcinogenesis [29,30,31,32,33,34,35].

**Figure 1:**
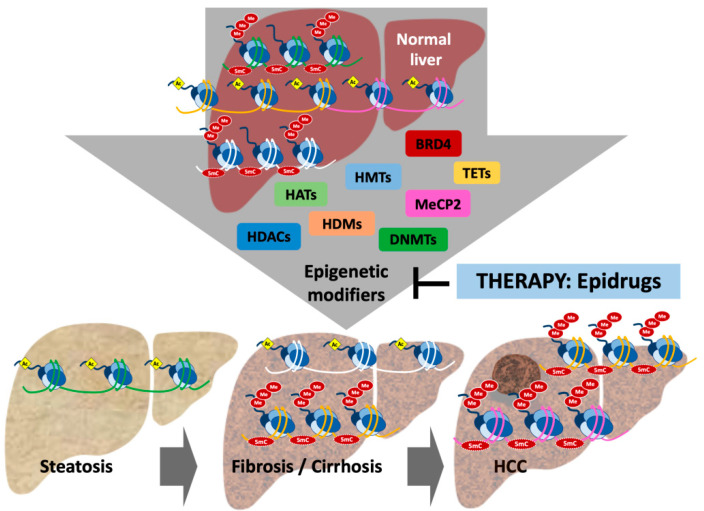
Epigenetic mechanisms control open and closed chromatin states, contributing to regulate gene expression. This drawing represents epigenetic changes described in the progression of chronic liver disease (see main text) such as DNA hypermethylation of tumor suppressor genes and histone acetylation of oncogenes. These changes are associated with the activity of different epigenetic modifiers, including histone methyltransferases (HMTs), histone demethylases (HDMs), histone acetyltransferases (HATs), histone deacetylases (HDACs), DNA methyltransferases (DNMTs), the ten-eleven-translocation (TETs) family of methyl-DNA dioxygenases, and the readers bromodomain-containing protein 4 (BRD4) and methyl-CpG-binding protein 2 (MeCP2). These enzymes and protein adaptors add, remove, or read the epigenetic marks representing new therapeutic targets for a variety of inhibitors known as epidrugs.

Together with the genetic landscape of mutations, multiple works have linked different changes in gene expression involved in the pathogenesis and progression of CLD with the rewiring of the epigenomic landscape [5,6,36] (Figure 1). Furthermore, multiple observations demonstrate the existence of an interplay between genetic and epigenetic alterations in liver cancer. On one hand, epigenetic alterations are influenced by genetic changes such as mutations in epigenetic modifiers [37], as we will discuss below. On the other hand, and reciprocally, there is an uneven distribution of somatic mutations that depend on the epigenetic context [38]. This would imply that cancer genome architecture depends on both precancerous and somatic epigenetic features.

The role of ncRNAs, more specifically miRNAs and lncRNAs, in hepatocarcinogenesis has been extensively reviewed elsewhere [39,40,41,42,43,44]. Regarding histone epigenetic marks, changes have been reported when comparing diseased with normal liver tissue, different stages of disease progression, or in correlation with disease aggressiveness [45,46,47,48]. For instance, the trimethylation of lysine 27 on histone H3 (H3K27me3), a transcription-suppressive histone mark incorporated by enhancer of zeste homolog 2 (EZH2), was detected by immunohistochemistry in HCC tissue samples in correlation with vascular invasion and predicting shorter survival time [45]. Interestingly, H3K27me3-enriched regions include a set of genes coding for transcription factors crucial for normal liver homeostasis, including NR1H4, HNF1A, HNF4A, and KLF9 [49]. More recently, increased and decreased levels of histone 3 at lysine 27 acetylation (H3K27ac), an activation of transcription mark, have been identified at overexpressed oncogenes and downregulated tumor suppressor genes, respectively, in livers from both non-alcoholic steatohepatitis (NASH) and HCV advanced fibrotic patients. Interestingly, these epigenetic and transcriptional changes were associated with the risk of HCC development [48] (Figure 1) (Table 1).

The hepatic DNA methylome has been extensively studied in different cohorts of patients with liver disease (Table 1). A marked depletion of 5hmC genomic content has been described in HCC being highly correlated with tumor stage [50]. Both genome-wide hypomethylation and region-specific hypermethylation are present not only in HCC but also in preneoplastic conditions [51,52,53]. Moreover, CpG methylation signatures have been associated with the progression of HVB-related liver disease [54], NAFLD fibrosis [55,56] and cirrhosis [57], and the poor survival of HCC patients [58]. DNA methylation changes repressing hepatic HNF4A-dependent gene expression have been associated with the loss of hepatocellular functions observed in patients with alcoholic hepatitis [59]. In agreement with this, together with the epigenetic silencing of tumor suppressor genes such as CDKN2A, HCC tissues display specific hypermethylation in genes related to hepatic differentiation, stem cell maintenance, and targets of the Polycomb repressive complex [29]. 

Genome-wide DNA methylation analyses comparing alcohol versus viral (HCV and HBV) [52] and NASH versus viral (HCV and HBV) [53] precancerous and HCC tissues revealed the existence of common as well as etiology-specific hepatocarcinogenesis methylome signatures. These studies demonstrate the impact of environmental factors such as viruses and diet in the pathological reshaping of chromatin, and thus in the regulation of gene expression. In this context, a recent study demonstrated the specific effect of dietary fructose intake in the modulation of multiple epigenetic mechanisms involved in the disruption of metabolic pathways underlying the pathogenesis of NAFLD [60]. Importantly, this epigenetic reprogramming can also occur in utero, and experimental evidence shows that long-term Western-style diet consumption affects offspring susceptibility to liver disease [61]. Given the reversibility of the epigenetic marks, maternal dietary interventions before pregnancy could represent an important preventive strategy to reduce obesity and NAFLD development in children [62]. However, it should be noted that diet-induced methylation changes in the liver can affect the expression of genes implicated in de novo and maintenance DNA methylation, such as DNMTs and TETs, and that these changes persist even after reverting to a normal chow diet [63]. Animal studies have also suggested the existence of transgenerational epigenetic inheritance in the development of liver disease, more specifically of liver fibrosis [64,65], where DNA methylation, histone PTMs, and ncRNA are major determinants [36]. At any rate, these aspects need validation in humans. 

Overall, the accessibility of DNA to the transcriptional machinery to activate or repress gene expression depends on the correct combination of all the epigenetic marks previously mentioned. Moreover, an important crosstalk between all the different epigenetic mechanisms exists [66,67,68]. For instance, the dysregulated expression of lncRNAs and miRNAs has been associated with changes in DNA methylation both in HCC and liver fibrosis, and on the contrary DNA methylation and histone PTMs depend on the activity of lncRNAs and miRNAs [39,69]. In fact, many of the altered epigenetic footprints identified in the damaged liver and described above are associated with changes in the expression of the corresponding epigenetic modifiers [70]. This has been demonstrated for key epigenetic writers and erasers such as EZH2 [71], DNMT1 and DNMT3 [72,73], the euchromatic histone-lysine methyltransferase 2 (EHMT2 or G9a) [73], TETs [13], and histone deacetylases (HDACs) [74]. Likewise, changes in the expression of epigenetic readers, such as the bromodomain-containing protein 4 (BRD4) [75] and methyl-CpG–binding protein 2 (MeCP2) [76], can potentiate the effect of the corresponding altered epigenetic marks. Again, and as an evidence of the genetic–epigenetic interplay, many of the changes in the expression or activity of the epigenetic modifiers observed in HCC can be associated with the presence of genetic mutations in those genes [37,77,78,79]. In fact, mutations in the mixed lineage leukemia (MLL) histone methyltransferase family and the adenosine triphosphate (ATP)-–dependent nucleosome remodeling enzymes (ARID) are among the most frequently described in HCC [80,81]. Moreover, aberrant epigenomes can also appear in the absence of epigenetic mutations by oncogenic mutations affecting upstream signaling pathways [82].

Liver homeostasis, and consequently disease development, depends on the interaction between multiple cell types. Recent genomic and transcriptomic studies performed at the single-cell resolution level have provided relevant discoveries in the hepatology field [83]. Bearing in mind that the epigenome is a cell-specific characteristic, the analysis of epigenetic changes at the single-cell level, including cancer, stromal, and immune cells, will significantly contribute to a better understanding of the hepatocarcinogenesis process, the development of more effective therapies, and the improved management of patients with liver disease. 

**Table 1:** Summary of epigenetic biomarkers found in tissue and liquid biopsies for liver disease diagnosis and prognosis.

**Epigenetic Biomarkers in Tissue Biopsy**				
**Epigenetic Alteration**	**Potential Use**	**Liver Disease**	**Affected Gene/Region**	**Reference**
DNA methylation	Diagnosis	HCC	Global 5hmC levels	[50]
			Differentially methylated CpGs	[51,52,53,58]
	Prognosis	HCC	Global 5hmC levels	[50]
			Differentially methylated CpG sigantures	[51,58]
		Fibrosis/Cirrhosis		
		Mix etiology	Differentially methylated CpG sigantures	[52,53,54,57]
		NAFLD/NASH	Differentially methylated CpG sigantures	[53,55,56]
		HBV/HCV	Differentially methylated CpG sigantures	[54,84]
		Alcohol	Differentially methylated CpG sigantures	[56,59]
Histone modifications	Prognosis	HCC	H3K27me3 at transcription factors crucial for liver homeostasis	[45]
		Fibrosis (mix etiology)	H3K27ac at specific oncogenes and TS	[48]
**Epigenetic Biomarkers in Liquid Biopsy**				
**Epigenetic Alteration**	**Potential Use**	**Liver Disease**	**Affected Gene/Region**	**Reference**
DNA methylation	Diagnosis	HCC	CDKN2A (P16)	[85]
			RASSF1A	[86,87]
			VIM	[88]
			FBLN1	[88]
			RGS10, ST8SIA6, RUNX2 and VIM	[89]
			cg04645914, cg06215569, cg23663760, cg13781744, and cg07610777	[90]
			HOXA1, EMX1, AK055957, ECE1, PFKP and CLEC11A	[91]
			GSTP1 and RASSF1A	[92]
			32-gene based 5hmC marker	[93]
			SEPT9	[94,95]
			BMPR1A, PSD, ARHGAP25, KLF3, PLAC8, ATXN1, Chr 6:170, Chr 6:3, ATAD2, Chr 8:20	[96]
	Prognosis	HCC	SEPT9	[95]
			SH3PXD2A, C11orf9, PPFIA1, Chr 17:78, SERPINB5, NOTCH3, GRHL2, TMEM8B	[96]
			Global 5hmC levels	[97]
			TFPI2	[98]
	Prognosis	Liver fibrosis	PPARγ	[99]

## 3. Epigenetic Biomarkers in Liquid Biopsy: Methylated ctDNA for Liver Disease Diagnosis and Prognosis

**Figure 2:**
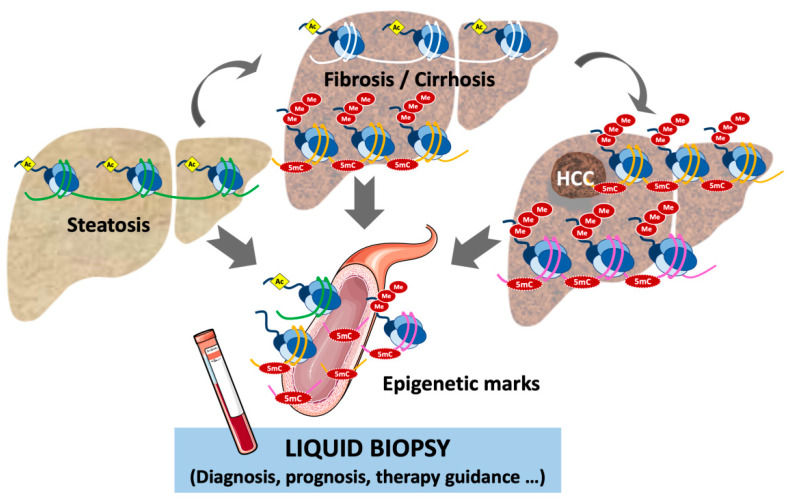
The epigenetically marked DNA and chromatin are released from the damaged liver to the bloodstream. Therefore, the identification of disease-specific epigenetic biomarkers in the plasma or liquid biopsy represents a non-invasive tool for the early detection of fibrosis or diagnosis of HCC and the prognosis and therapeutic guidance of patients.

Liquid biopsy refers to the analysis in the bloodstream or other body fluids of material derived from the tumor, or in some cases the damaged tissues, including cell-free DNA (cfDNA) and RNA, proteins, circulating tumor cells (CTCs), metabolites, and extracellular vesicles [100,101,102] (Figure 2). Circulating cfDNA and CTCs are the cornerstone analytes of liquid biopsy, providing the genetic and epigenetic profiles of the cells of origin, and importantly recapitulating the complex and heterogeneous molecular pathogenesis of tumors such as HCC [103,104,105]. Significant advances have been made in the technology and protocols used to isolate and evaluate the analytes. However, the identification of specific and sensitive biomarkers for patient diagnosis, prognosis, monitoring, and therapy guidance remains a challenge [101]. In the context of liver diseases, where a large proportion of tumors develops on a background of liver cirrhosis, the identification of reliable biomarkers [33] and the use of liquid biopsy represents a promising tool for the screening and early detection of HCC. Moreover, liquid biopsy could overcome the intra- and inter-tumor genetic and epigenetic heterogeneity of HCCs, helping with the therapeutic management of HCC patients.

Many efforts have been made to identify biomarkers from the mutational profiling of ctDNA [106]. However, although the proof-of-concept has been made, the paucity of hotspot mutations in HCC and the presence in the circulating DNA of mutations originated from clonal hematopoiesis rather than from tumor cells giving rise to false-positive cases, among others, have limited the applicability in the clinic of liquid biopsy tests based on mutation biomarkers [101,104,105,107].

Multiple studies have shown that the screening of specific epigenetic modifications, in particular the methylation profiling of ctDNA, has great potential across different clinical scenarios in the management of HCC [104,105] (Figure 2). As mentioned above, DNA methylation patterns are cell-type-specific, are highly stable as covalent modifications, and are aberrantly and specifically altered in cancer cells [2,108]. Moreover, it has been known for some time that DNA methylation changes in HCCs, such as the hypermethylation of CpG-rich promoter sequences of tumor suppressor genes, are consistently detected in the ctDNA of HCC patients [109], even several years before the clinical diagnosis of the tumor [86]. 

In fact, hypermethylation of the *CDKN2A* gene encoding for the cell cycle regulator tumor suppressor gene p16 has been assayed as a single methylated ctDNA biomarker for the detection of HCC, although with very variable results [85]. Other single methylated ctDNA biomarkers tested in case–control studies include genes encoding for Ras association domain family 1A (*RASSF1A*) [86,87], vimentin (*VIM*) [88], fibulin 1 (*FBLN1*) [88], and tissue factor pathway inhibitor-2 (TFPI2) [98]. More recently, methylated septin 9 (*SEPT9*) gene, the biomarker used in the first blood-based test commercialized for the early detection of colorectal carcinoma, has demonstrated high accuracy (area under the receiver operating curve, AURC, of 0.94) to diagnose HCC in two independent cohorts [94] and to predict long-term survival of HCC patients [95]. *SEPT9* is a key regulator of cell division implicated in liver carcinogenesis [110], which is silenced by promoter hypermethylation not only in HCC [58] and colorectal cancer, but also in other tumors such as head and neck squamous cell carcinoma and breast cancer [111,112]. This lack of tumor specificity could limit its applicability, and further developments are needed to allow the identification of hepatocyte-specific methylation patterns. To trace the origin of ctDNA, new strategies could include, for instance, the identification of liver-associated cell-free DNA-end signatures [113], liver-specific nucleosome footprints [114], or HCC-specific cell-free 5hmC signatures [97]. 

Test performance could be improved by implementing panels of ctDNA-methylated markers. As summarized in Table 1, using different approaches multiple groups have identified the high sensitivity and specificity of four [89], five [90], six [91], or ten [96] plasma ctDNA-methylated markers for HCC diagnosis or eight [96] plasma ctDNA-methylated markers for HCC prognosis prediction. HCC recurrence following tumor resection was also detected up to 9 months before MRI diagnosis by monitoring *GSTP1* and *RASSF1A* ctDNA methylation in the urine of patients [92]. All these studies highlight the great potential of ctDNA methylation profiling for the management of HCC; however, the diversity of the panels described suggests that validation studies are required. Those studies should also include samples from patients with early-stage HCCs and from longitudinal follow-up of cirrhotic patients. 

Interestingly, 5hmC signatures can be also monitored in circulating HCC-specific cfDNA, which could also be applied to track tumor stage [97]. Accordingly, a recent non-invasive 32-gene based 5hmC marker panel has been developed and tested for the early detection of HCC [93,115].

Epigenetic aberrations contribute to the pathogenesis and progression of chronic liver disease [5,65], and changes in DNA methylation have been described at different stages, including hepatitis, fibrosis, and cirrhosis [56,57,59]. Importantly, the identification of stage-specific changes in ctDNA would facilitate the monitoring of disease progression and the early diagnosis of tumors. In this sense, the detection in ctDNA of two CpG sites differentially methylated at the promoter of the peroxisome proliferator-activated receptor γ gene (*PPARγ*) has been proposed to stratify liver fibrosis in patients with NAFLD [99]. 

Overall, the proof-of-concept confirming the power of liquid biopsies as non-invasive tools for the management of patients with CLDs has been made. However, the results also suggest that the reliability and utility of liquid biopsy tests most likely rely on the use of multi-marker analyses. Further studies are needed to evaluate the performance of combination of methylated cfDNA biomarkers with ctDNA mutations and/or protein biomarkers in nucleosomes [116], extracellular vesicles [117], or CTCs [118], together with markers of tissue of origin [113,114]. Moreover, the combination of these emerging epigenetic biomarkers with currently used serological markers can be envisioned to be used to improve HCC diagnosis, patient stratification, and evaluation of treatment response. In this regard, several studies have shown increased sensitivity when their identified epigenetic liquid biopsy biomarkers are combined with AFP determination [93,95,98]. The presence and levels of naïve viral RNA and DNA [119], its integration in cellular DNA, or even epigenetic modifications of viral sequences [84] along the hepatocarcinogenesis process could also be combined. Furthermore, large clinical trials as well as standard protocols for the isolation of analytes and the identification of biomarkers are needed.

## 4. Harnessing Epigenetic Alterations for Therapy

As reviewed above, multiple findings demonstrate that epigenetic dysregulation plays a role in the pathogenesis and progression of NAFLD, fibrosis, cirrhosis, and hepatocellular carcinoma. Given the plasticity and reversibility of the epigenetic modifications, they represent promising therapeutic targets. Indeed, over the last years DNA hypomethylating agents and histone deacetylase inhibitors (HDACi) have been approved or are being tested for the treatment of hematologic malignancies and solid tumors [4,70,120,121]. Moreover, recent results have demonstrated that the targeting of epigenetic writers (DNMTs and histone methyltransferases, HMTs), readers (BRDs), and erasers (HDACs and lysine demethylases, KDMs) can synergize with immunotherapy, increasing the immune recognition of tumor cells [5,122,123,124].

We have recently reviewed the current advances in the epigenetic treatment of HCC [5]. Multiple studies have explored different first-generation DNMT inhibitors (DNMTi), and among them decitabine showed beneficial clinical response and favorable toxicity profiles in patients with advanced HCC in a phase I/II clinical trial [125]. A second-generation DNMTi is guadecitabine (SGI-110), in which decitabine is linked to deoxyguanosine to confer additional stability and a longer half-life due to protection from deamination. Guadecitabine has demonstrated very promising anti-tumoral results in preclinical studies [126,127]. Mechanistically, SGI-110 re-induced the expression of tumor suppressor genes and demethylated gene body regions in pro-tumorigenic genes, including the HMT EZH2, downregulating their expression. Importantly, SGI-110 robustly reactivated the expression of epigenetically silenced endogenous retroviruses, enhancing tumor immunogenicity that can be harnessed to improve immune checkpoint inhibitor sensitivity in vivo [128]. Accordingly, two clinical trials are testing the activity of SGI-110 in patients with advanced HCC when administered alone or in combination with the immunotherapeutic agent durvalumab (anti-PDL1). Peer-reviewed publication of the results is awaited. 

Regarding HDACi, the pan-HDACi belinostat (PXD-101) showed tumor stabilization and was generally well-tolerated in a phase I trial in patients with unresectable HCC [129]. More recently, its combination with immune checkpoint inhibitors increased their efficacy in an animal model of HCC [130]. The SHELTER phase I/II clinical trial demonstrated positive results of the combination of the HDACi resminostat with sorafenib as second-line therapy in patients with advanced HCC [131]. HMTi, in particular EZH2 inhibitors, have been tested in preclinical studies. GSK126 enhances natural killer cell–mediated eradication of HCC cells [132]; however, in another study the combination of GSK126 with decitabine promoted hepatoma progression in an orthotopic HCC model in mice, due to an impaired antitumorigenic T cell response [133]. Two other EZH2 inhibitors have demonstrated activity in preneoplastic models. 3-Deazaneplanocin A (DZNep) [134] and GSK503 [135] attenuate liver fibrosis in the murine model of carbon tetrachloride (CCl_4_), and GSK503 is also effective in the bile duct ligation (BDL) model, inhibiting the activation of hepatic stellate cells. 

Benefits of combined therapies, using separate molecules or multitargeted molecules with two biologically active units, include synergistic effects and prevention of drug resistance. As a multitarget epigenetic therapeutic strategy, our group has developed the inhibitor CM-272 combining inhibitory activities for the HMT G9a and DNMT1, both enzymes being coordinatively dysregulated during the progression of liver disease [73]. The dual inhibitor CM-272 was able to restore the differentiated phenotype of HCC cells and to inhibit tumor growth in vivo even in the presence of fibrogenic stroma [73]. Moreover, CM-272 counteracts the pro-fibrogenic metabolic reprogramming of HSC and inhibits CCl_4_-induced fibrogenesis in vivo [136]. The efficacy of CM-272 has also been recently demonstrated in cholangiocarcinoma, a type of hepatobiliary tumor for which no effective systemic therapies exist [137].

As mentioned above, in patients with NASH and HCV-related advanced fibrosis, HCC risk is associated with increased levels of H3K27ac in hepatic chromatin [48]. Accordingly, the inhibition of the H3K27ac reader, the bromodomain-containing protein 4 (BRD4), with the small-molecule JQ1 reverted the bad prognosis-associated transcriptional program in HCC cells, and significantly reduced tumor burden in a mouse model of NASH-induced hepatocarcinogenesis [48]. Moreover, JQ1 prevented HSC activation and fibrosis development in the CCl_4_ mouse model of liver fibrosis [138].

Altogether, these results highlight the potential of epidrugs not only as anti-tumoral therapies, but also as agents able to boost antitumor immune responses [124] and to halt or reverse liver fibrogenesis, which is a key contributor to HCC development.

## 5. Conclusions

Evidence has demonstrated that epigenetic alterations, in response to environmental cues or genetic changes, play a critical role in the pathogenesis of CLD. In fact, specific epigenetic marks could be used as biomarkers to diagnose or predict CLD progression, and their identification in liquid biopsy represents a very promising strategy to aid in the management of CLD patients. Moreover, as epigenetic marks are dynamically and reversibly established, epigenome-targeted therapies are increasing the drug portfolio also for solid malignancies. Different epidrugs have been developed to modulate the activity of the pleiad of epigenetic writers, erasers, and readers. In particular, HDACi and DNMTi have been tested alone or in combination with immunotherapy to treat patients with different types of cancer, including HCC patients. Moreover, available data suggest that these strategies could be envisioned to prevent the progression of CLD. However, several aspects may challenge the success of epigenetic modulation. One important issue would be the lack of selectivity. These inhibitors are highly nonspecific, and upon systemic administration they can affect any cell type, as well as any gene in the cell under such epigenetic control regardless of its pathological role. Moreover, many of the targeted epigenetic modifiers have functions outside the chromatin. For instance, interference with the acetylation of other non-histone proteins such as the tumor suppressor gene P53 may have unexpected effects. Furthermore, given the crosstalk between different epigenetic mechanisms, targeting a specific mark could affect other modifications, and so far, this “network” effect is difficult to predict.

Altogether, further basic research is needed to improve our mechanistic understanding of the epigenetic processes and their interactions, their dysregulation in disease, and the cellular response to epigenetic therapies. This effort, together with well-designed clinical trials, will help to boost epigenetic-based personalized medicine.
